# Continuous Descending Modulation of the Spinal Cord Revealed by Functional MRI

**DOI:** 10.1371/journal.pone.0167317

**Published:** 2016-12-01

**Authors:** Patrick W. Stroman, Rachael L. Bosma, Andreea I. Cotoi, Roxanne H. Leung, Jennifer Kornelsen, Jane M. Lawrence-Dewar, Caroline F. Pukall, Roland Staud

**Affiliations:** 1 Centre for Neuroscience Studies, Queen’s University, Kingston, Ontario, Canada; 2 Department of Biomedical and Molecular Sciences, Queen’s University, Kingston, Ontario, Canada; 3 Department of Physics, Queen’s University, Kingston, Ontario, Canada; 4 Dept of Radiology, University of Manitoba, Winnipeg, Manitoba, Canada; 5 Thunder Bay Regional Research Institute, Thunder Bay, Ontario, Canada; 6 Department of Psychology, Queen’s University, Kingston, Ontario, Canada; 7 Department of Medicine, University of Florida, Gainesville, Florida, United States of America; University of Modena and Reggio Emilia, ITALY

## Abstract

Spontaneous variations in spinal cord activity may arise from regulation of any of a number of functions including sensory, motor, and autonomic control. Here, we use functional MRI (fMRI) of healthy participants to identify properties of blood oxygenation-level dependent (BOLD) variations in the spinal cord in response to knowledge that either a noxious stimulus is impending, or that no stimulus is to be expected. Expectation of a noxious stimulus, or no stimulus, is shown to have a significant effect on wide-spread BOLD signal variations in the spinal cord over the entire time period of the fMRI acquisition. Coordination of BOLD responses between/within spinal cord and brainstem regions are also influenced by this knowledge. We provide evidence that such signal variations are the result of continuous descending modulation of spinal cord function. BOLD signal variations in response to noxious stimulation of the hand are also shown, as in previous studies. The observation of both continuous and reactive BOLD responses to emotional/cognitive factors and noxious peripheral stimulation may have important implications, not only for our understanding of endogenous pain modulation, but also in showing that spinal cord activity is under continuous regulatory control.

## Introduction

The spinal cord is often portrayed as a passive relay point for neural signaling, but its function is far more complex and is regulated by a number of brainstem regions [1]. In the absence of a stimulus or task, fluctuations in neuronal activity in the spinal cord have been detected by means of functional MRI, and are thought to provide important information about networks of regions across the CNS [2–4]. However, due to methodological challenges very few “resting-state” fMRI studies have been carried out in the spinal cord and no specific networks have been identified. It has been speculated that the source of resting-state fluctuations (RSF) may be linked to brain resting-state networks, muscle coordination, and/or descending regulation of sensory signaling [2–4]. Smith and Kornelsen [5] showed variations in blood oxygenation-level dependent (BOLD) fMRI signal in the cord when participants viewed negative, positive, and neutral pictures from the International Affective Picture System (IAPS) database. These results suggest that fluctuations in neuronal activity in the spinal cord can be related to emotional responses.

Tonic input from brainstem regions, including the periaqueductal gray matter (PAG) and rostral ventromedial medulla (RVM), regulates spinal cord sensory/pain signaling [1]. Thus the “baseline” neuronal activity in the spinal cord is not an “inactive” state. This conclusion has been supported by the results of several studies, which demonstrate negative responses (i.e. decreases in activity) in some spinal cord and brainstem regions in response to heat/pain stimuli [6–8]. The PAG-RVM pathway has been shown in animal studies to produce analgesia in threatening situations, and analgesia is inhibited if the environment is perceived to be safe [9, 10]. These studies indicate that neurons in the RVM exert global control over pain transmission [11]. We therefore propose that variations in the descending input, absent of a stimulus, could explain observed resting-state BOLD signal changes and sources of signal variance which are not accounted for by responses to stimulation/task paradigms, nor by physiological noise [12]. Analyses of time-series data obtained in a number of previous spinal cord fMRI studies in our lab [6, 8, 13–16] support the hypothesis that systematic BOLD signal variations can occur in the absence of a stimulus. Participants in these studies were familiarized with the study paradigm and could easily anticipate whether or not noxious stimuli were impending (threat), or had passed (safety). We therefore hypothesize that systematic variation of BOLD signals in the spinal cord can arise as a result of the participants’ cognitive/emotional state related to the perceived threat that could occur with expectation of a noxious stimulus, or perceived safety with expectation that a stimulus will not be applied. Because this study therefore involves manipulation of the emotional/cognitive state, it cannot be considered a “resting-state” study, but the results can provide insight into possible sources of resting-state fluctuations in the spinal cord and brainstem. The results of this study also have important implications for our understanding of how emotional and/or cognitive factors influence pain processing networks.

## Methods

This research was reviewed and approved by the Queen's University Health Sciences Research Ethics Board (protocol number CNS-015-15). All participants provided fully informed written consent prior to participating. A total of 17 healthy participants (13 female, 4 male, aged 22 ± 3 years, range 19–31 years), were recruited from the local community, and provided informed consent. The required number of participants was estimated with a statistical power calculation based on a two-sample t-test, with the aim of discriminating responses between study conditions with values that differ by 1 standard deviation, at p < 10^−3^. Participants had no history of neurological disease, major medical illness or psychiatric disorder. All procedures were reviewed and approved by our institutional research ethics board.

### Experimental design

All participants underwent quantitative sensory testing and training, as described below, followed by an imaging session. Participants were first trained to use a standardized numerical pain scale (NPS) to rate the magnitude/intensity of their pain experience [17, 18]. The scale ranges from 0 to 100, in increments of 5, with verbal descriptors at intervals of 10: 0 –no sensation, 10 = warm, 20 = a barely painful sensation (i.e., pain threshold), 30 = very weak pain, 40 = weak pain, 50 = moderate pain, 60 = slightly strong pain, 70 = strong pain, 80 = very strong pain, 90 = nearly intolerable pain, and 100 = intolerable pain. A series of threshold and calibration tests were performed. All heat stimuli were applied to the skin via an MR-compatible, Peltier thermode (Medoc^®^, Ramat Yishai, Israel). The Medoc device was programmed to control the temperature while the thermode was held by one of the experimenters and applied manually to the skin as needed for the specific test or stimulus.

In order to allow comparisons with previous studies we employed a method of heat stimulation with temporal summation of second pain (TSSP) which has been found to yield robust BOLD responses in the brainstem and spinal cord in previous studies [13, 19, 20]. This stimulation method consists of repeatedly contacting the skin with a heated thermode for brief periods. As a result, the stimulus also includes a sensory component related to the repeated contacts. The heat stimuli were applied to the right palm, on the skin overlying the thenar eminence, to correspond with the 6^th^ cervical dermatome. The temperature was calibrated for each participant to evoke approximately the same pain intensity (50 on the 100-point scale), which is described as “moderate pain” on the rating scale.

In order to familiarize participants with the study procedures, and to calibrate the stimulation temperature, the heated thermode was contacted with the skin 8 times, for 1.5 seconds each, with onsets every 3 seconds. For the series of contacts the experimenter was guided by audio cues (imperceptible to the participant) to indicate the duration and timing onset of each contact. Participants were instructed to rate their pain from each contact. This process was repeated at 46°C, 50°C, 44°C, and 48°C, separated by at least 2 minutes of rest. The reported pain ratings were used to determine the temperature needed to achieve a sensitivity-adjusted final rating of 50 ± 10 NPS units on the scale.

**Fig 1:**
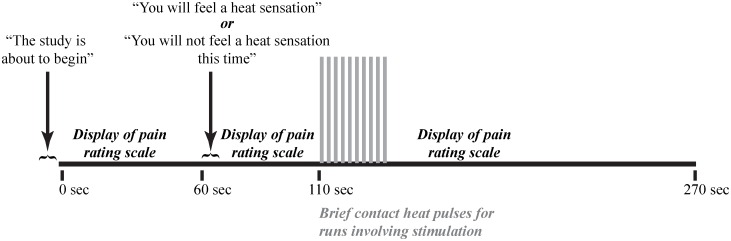
The task paradigm for the no-stimulation and heat stimulation conditions. The temperature of stimulation was calibrated for each individual to produce a moderate pain rating (50 on a 100-point scale) for the last heat contact of the paradigm. For the heat stimulation condition, the heat stimuli were applied every 3 seconds, for 1.5 seconds.

Participants then underwent mock fMRI scanning to become familiar with the scanning environment. Participants lay down on the mock MRI scanner bed and underwent the experimental protocol as it would be presented in the subsequent MRI sessions, including hearing recordings of the scanner sounds during imaging. They viewed a rear-projected screen (via a mirror) on which notifications were displayed when a new run was about to start, and whether or not the run would include heat stimulation. As in the earlier tests involving heat stimulation, the thermode was heated to the calibrated temperature and was applied in a series of 10 brief (1.5 second) contacts to the thenar eminence of the right hand at an inter-stimulus interval (onset to onset) of 3 seconds (0.33 Hz) (Fig 1). Contrary to the earlier tests, participants were instructed to silently rate their pain to each heat contact in an effort to have them remember their pain rating at the time it was experienced, rather than rate a memory of their pain experience. At the end of each run they were asked to report their rating of the first and last heat contacts. This replicated the procedures used to limit movement during the actual fMRI acquisitions after the training stage was complete.

### FMRI paradigm

The stimulation paradigms used during subsequent fMRI sessions were similar to those practiced during the mock fMRI session. Each run lasted 4 minutes and 30 seconds, regardless of whether or not heat stimulation was applied. At the beginning of each run, the participant was informed, via the rear-projection display, that a new run was beginning, but the participant did not know if heat stimulation would be applied (Fig 1). After one minute, the participant was informed that either they would feel a heat sensation (“stimulation” condition), or that no heat would be applied (“no-stimulation” condition). This study design is intended to manipulate the emotional/cognitive state during each acquisition, by producing a perception of “threat” when the participant expects a noxious stimulus, and a perception of relative “safety” when no stimulus is expected, or after the stimulus has been applied. In runs with no stimulus, the fMRI time-series acquisition was simply continued until the end of the run. In runs involving the heat stimulus, the stimulation period began 50 seconds later (1 minute 50 seconds from the start of the run), and lasted 30 seconds, as in the training session. Again, stimulation consisted of brief heat contacts, 1.5 seconds in duration, applied 10 times with onsets every 3 seconds. The heat contacts were applied by one of the experimenters, to the skin overlying the right thenar eminence, with timing guided by audio cues perceptible only to the experimenter via head-phones. The stimulation period was followed by 2 minutes and 10 seconds of data acquisition during the subsequent state after the stimulation was completed. Five runs of each of the stimulation and no-stimulation conditions were implemented in a random, counterbalanced order. At the end of each run involving heat stimulation, participants were asked to report their ratings to the first and last heat contacts.

### FMRI data acquisition

All image data were acquired using a 3T whole-body MRI system (Siemens Magnetom Trio; Siemens, Erlangen, Germany). Participants were positioned supine and were supported by padding as needed for comfort and to restrict bulk body movement, and entered the MRI system head-first. Initial localizer images were acquired in three planes as a reference for slice positioning for subsequent fMRI studies. Data were acquired using a posterior head coil (6 elements), a posterior neck coil (3 elements), and, depending on the size of the participant, the upper 3 elements of a phased-array spine receiver coil (6 x 3 array). A body coil was used for transmitting radio-frequency (RF) excitation pulses. In order to obtain optimal spatial fidelity in the brainstem and spinal cord, as well as BOLD sensitivity, fMRI data were acquired using a half-Fourier single-shot fast spin-echo sequence [12]. A 3D volume that spanned from the T1 vertebra to above the thalamus was imaged repeatedly to produce each fMRI time-series. Nine sagittal slices were acquired contiguously with a repetition time (TR) of 6.75 sec/volume, an echo time of 76 msec to optimize the T_2_-weighted BOLD sensitivity, a 28 × 21 cm field-of-view with 1.5 × 1.5 × 2 mm^3^ resolution. A total of 200 volumes were acquired for each condition (over 5 repeated runs). The image quality was enhanced by means of spatial suppression pulses anterior to the spine to reduce motion artefacts caused by breathing, swallowing, etc., and motion compensating gradients in the head-foot direction.

### Data analysis

#### Data pre-processing

The 3D spinal cord/brainstem functional imaging data were analyzed with custom-made software written in MATLAB^®^. Image data were first converted to NIfTI format, and were co-registered to correct for bulk motion using the non-rigid 3D registration tool in the Medical Image Registration Toolbox (MIRT) [21, 22]. The coregistration method has been shown previously to account for a significant component of physiological motion, and from this step we also extracted parameters describing the bulk motion during each acquisition [12]. The images were then resized to 1 mm cubic voxels and spatially normalized using custom-made automated normalization software written in MATLAB, as described previously [12, 13]. Spatial normalization mapped the image data from each participant to our established 3D anatomical template, generated from images of 356 participants [13, 23]. The mapping to the normalized template was also fine-tuned using the MIRT toolbox [22]. Brainstem and spinal cord regions are also defined in the normalized space in a predefined anatomical map.

#### Connectivity analysis (correlation)

Coordination of fMRI signal variations across spatially-distinct regions was tested by means of seed-to-voxel connectivity analyses. The “seed” is a selected set of voxels in a specific anatomical region. Seed regions were defined as 2 x 2 x 2 mm cubic volumes in the right and left side of the cord, in dorsal and ventral gray matter regions, at the approximate center of the 6^th^ cervical spinal cord segment. Correlation between the time-series fMRI data in the seed region and in any other voxel implies that the regions have common BOLD signal variations, and therefore common timing of input signaling.

Data were first pre-processed with motion correction and spatial normalization, as described above, and signal intensity variations matching the extracted motion parameters were removed by means of regression. The time-series data were converted to z-scores by subtracting the average intensity, and dividing by the standard deviation, on a voxel-by-voxel basis. Data were concatenated across runs/participants to produce one large time-series for each seed region, or each voxel, and the seed-to-voxel temporal correlation was then computed for all voxels. Correlation values were also converted to z-scores in order to determine the significance (p-value). Voxels with significant correlation with the seed region (p < 0.05, Bonferroni corrected) were identified, and the average fMRI time-series across these voxels was determined.

#### Structural equation modeling

FMRI time-series data from the brainstem and spinal cord were also analyzed by means of structural equation modeling (SEM), with custom made software written in MATLAB [23–25]. SEM reveals coordinated BOLD responses across regions, allowing for the fact that input to one region is often the sum of signaling from multiple other regions [26, 27]. The underlying concept is that the BOLD signal intensity time-course in each region can be expressed as a linear combination of the BOLD responses in other regions, and the weighting factors for the linear combination reflect the “connectivity strengths” [26]. The SEM analysis requires a pre-defined anatomical model of plausible connections between regions and was based on our reference template described above. Contributions to the BOLD signal variations observed in the spinal cord dorsal horn were investigated by determining the input contributions from the dorsal reticular nucleus (DRt) and regions of the RVM; the nucleus gigantocellularis (NGc) and the nucleus raphe magnus (NRM). These regions were selected for their expected importance in providing direct modulatory input signaling to the spinal cord.

Data used for the SEM analysis were pre-processed as described above, including subtraction of components matching the motion parameters, and were averaged across runs/participants to represent the consistent time-series responses in each voxel. Anatomical regions including the NRM, NGc, DRt, and the right dorsal region of the spinal cord within the 6^th^ cervical segment (C6), were identified from our 3D anatomical map, and time-series data were extracted for each voxel. The right dorsal region at C6 was selected because it is expected to be the site of input from the periphery when the C6 dermatome on the right hand is stimulated. The voxels within each region were then divided into 7 sub-regions based on the time-series data properties, by means of k-means clustering. The purpose of this step was to use the time-series properties to group voxels with significant BOLD responses separately from non-responding voxels, or those that are dominated by physiological noise. All sub-regions were included in the analysis, and all possible combinations of regions were tested. The results provide the linear weighting factors (i.e. the relative contribution of each input to a region), and the sub-regions of the NRM, NGc, DRt and cord DH, that yield the best fit to the measured data.

#### Dynamic connectivity

SEM analysis results demonstrate the overall coordination between regions but the relationships may vary across periods of the stimulation paradigm. Dynamic variations in connectivity were therefore assessed by computing the correlation between time-course responses with a sliding window spanning 41 seconds (i.e. 6 volumes) throughout the 270 sec paradigm. The choice of time-span was made in an effort to balance having a sufficient number of volumes to detect correlations, while also being adequately short to reveal transient effects. Average time-series responses from regions identified by the SEM analysis described above were analyzed to investigate the contributions from each region (NRM, NGc, and DRt) to the BOLD signal variations in the spinal cord.

## Results

Temperatures used for stimulation averaged 48.0 ± 2.0°C across the 17 participants, and pain ratings had average values of 51.3 ± 13.5 across 85 fMRI runs. Pain ratings were weakly negatively correlated with the stimulation temperature (R^2^ = 0.05, p = 0.017) but the variation had a greater dependence on the run number across the 5 times the stimulation paradigm was applied (R^2^ = 0.65), than on the temperature (R^2^ = 0.16).

### Connectivity results

**Fig 2:**
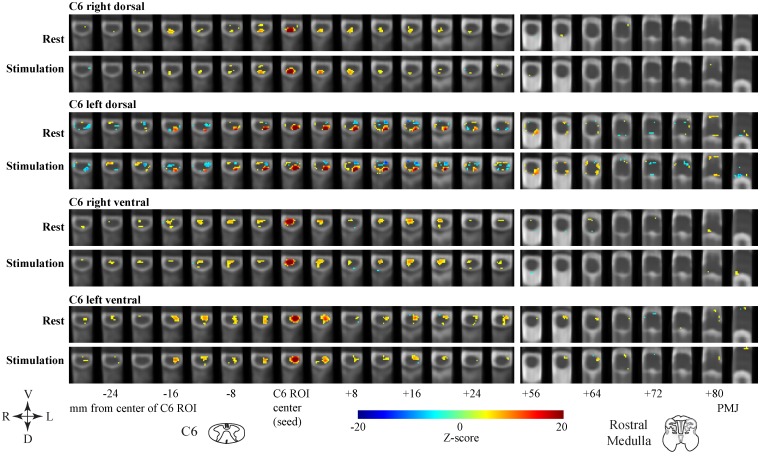
Connectivity between voxels and selected seed regions. Anatomical locations of regions with time-courses that are significantly correlated with seed regions in the right/left and dorsal/ventral regions of C6, are shown in colour overlaid on gray-scale anatomical images. Each frame represents a 1 mm thick transverse slice, and slices are shown every 4 mm along the cord (horizontally in the figure) to depict the rostral-caudal range along the spinal cord, and a section of the medulla. The seed regions are identified by the highly correlated (dark red) voxels within the regions. Positions along the cord/brainstem are indicated relative to the seed region center, in millimeters.

Fig 2 shows maps of the voxels in the cervical spinal cord and brainstem, which have significant (p < 0.05, Bonferroni corrected) connectivity with 2 mm cubic seed regions in gray matter regions in four quadrants (right/left dorsal/ventral) of the spinal cord at C6. All seed regions are shown to be extensively connected with voxels in the same quadrant, over a range spanning approximately -20 mm to +28 mm from the seed centers, as well as within localized regions of the brainstem, in both no-stimulation and stimulation conditions. Voxels with significant connectivity to the right and left dorsal seed regions are demonstrated in ipsilateral ventral regions in both study conditions as well. Similarly, right and left ventral seed regions are connected to ipsilateral dorsal regions in some locations along the cord as well. It is notable that right- and left-ventral and left-dorsal seed regions have more connectivity with right dorsal voxels in the stimulation condition than in the no-stimulation condition. The left dorsal seed region has notable negative connectivity with ipsilateral ventral regions, and areas of the RVM.

**Fig 3:**
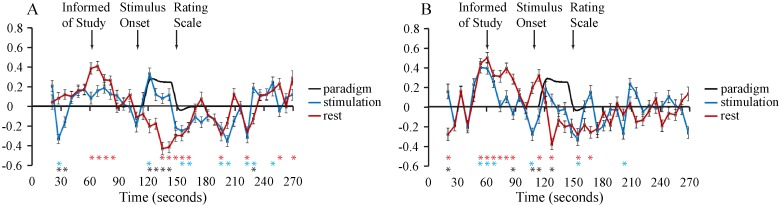
Average time-courses of spinal cord voxels which are significantly correlated with seed regions in (A) (left) the right-dorsal region of C6, and (B) (right) the left-dorsal region of C6. The voxels are as depicted in Fig 2. Signal intensity patterns obtained during stimulation conditions are shown in blue, and the no-stimulation condition is shown in red. Time-course data were converted to z-scores with mean values of zero. Error bars indicate the standard-error-of-the-mean across fMRI runs. Red and blue asterisks (*) indicate values which are significantly different than the mean value of zero, at p < 10^−4^, for no-stimulation and stimulation runs, respectively. Black asterisks indicate intensity values which are significantly different between the two study conditions, at p < 10^−4^. Times are indicated corresponding to when participants were informed of the study type, the start of the stimulation period (for studies with heat stimulation), and the time at which the rating scale was displayed in all studies.

Average signal intensity time-courses (z-scores) are shown in Fig 3 for voxels with significant connectivity to the seed regions in right and left dorsal regions of C6. Signal variations were inferred to be significant when intensity values deviated from the time-course average at p < 10^−4^, determined with a one-sample t-test. Intensity values between study conditions (no-stimulation vs stimulation) were also compared with an unpaired two-sample t-test, and were inferred to be significant at p < 10^−4^. A significant signal intensity response is apparent when the participants were informed of the type of study (stimulus, or no-stimulus) in all conditions/seed-regions, with the exception of the right DH when a stimulus was expected. During the period when the stimulus is applied there is a significant BOLD response in the right DH that closely corresponds with the expected response to the heat stimulus, and this response is apparent as well in the left dorsal region. During this same period the signal intensity decreases significantly in both the right and left dorsal regions in no-stimulation runs. The signal intensities are only significantly different between the two study types during the usual stimulation period on the right side, and just prior to this period on the left side. Finally, in the period following when the stimulus is applied, or would be expected, both the right and left regions have gradually increasing signal intensities in both the stimulation and no-stimulation runs. During this period the time-series responses in the right dorsal region are significantly correlated between the no-stimulation and stimulation runs (R = 0.65, p < 0.01). No other significant correlations were detected over this period across study types and right/left regions.

### SEM results

**Fig 4:**
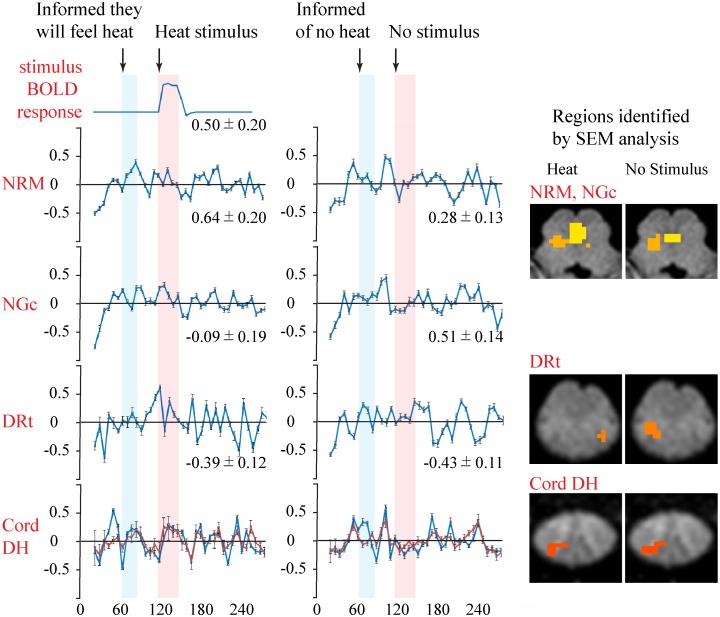
A structural equation modeling (SEM) analysis was used to investigate input signaling to the spinal cord dorsal horn (DH) in the 6^th^ cervical segment, from the nucleus raphe magnus (NRM), nucleus gigantocellularis (NGc), and the dorsal reticular nucleus (DRt). Results are shown for the sub-regions (right panel) which provided the best fit to the BOLD signal intensity variations in the spinal cord DH, when a stimulus was expected and applied, and when a stimulus was not expected and not applied (i.e. no-stimulation). The time-series responses in the identified regions are shown (left panels) for each study condition, as well as the SEM fit to the spinal cord time-series responses (red lines). Plotted values are the average over the region across runs/participants and error bars indicate the standard error of the mean (SE) across participants. The SEM weighting factors that were determined, and used for the fit, are indicated below each time-series plot (± SE). Periods are highlighted when the participants were informed of the study type, and when the stimulus was applied, or was not applied.

Structural equation modeling demonstrated significant contributions from the NRM, NGc, and DRt to the BOLD signal variations observed in the cord DH (6^th^ cervical segment), both when a stimulus was expected and applied, and when it was neither expected nor applied. The sub-regions that provide the best fit to the signal variations observed in the spinal cord DH in each study condition are demonstrated in Fig 4. These results show that when a stimulus is expected/applied the BOLD signal variations in the spinal cord DH can be expressed as a sum of inputs from the periphery (P), the NRM, and the left DRt: S_DH, Stim_ = (0.50 ± 0.20) S_P_ + (0.64 ± 0.20) S_NRM_ + (-0.39 ± 0.12) S_DRt_ (R^2^ = 0.42, p = 1.2 x 10^−5^).

This result indicates that a BOLD response matching the peripheral stimulation, plus input from the NRM and DRt, can together explain 42% of the variance in the cord DH during runs with a heat stimulus applied. When a stimulus was neither expected nor applied, the BOLD signal variations observed in the cord DH can be expressed as a sum of inputs from the NRM, right NGc, and the right DRt: S_DH, No-Stim_ = (0.28 ± 0.13) S_NRM_ + (0.51 ± 0.14) S_NGc_ + (-0.43 ± 0.11) S_DRt_ (R^2^ = 0.48, p = 1.5 x 10^−6^)

Input signaling modeled from BOLD signal variations in the NRM, NGc, and DRt, can together explain 48% of the variance in the cord DH during no-stimulation runs. Significance is inferred at a Bonferroni-corrected p < 0.05, to correct for multiple comparisons with a total of 2401 possible network connections (p_uncorrected_ < 2.1 x 10^−5^).

### Dynamic connectivity

**Fig 5:**
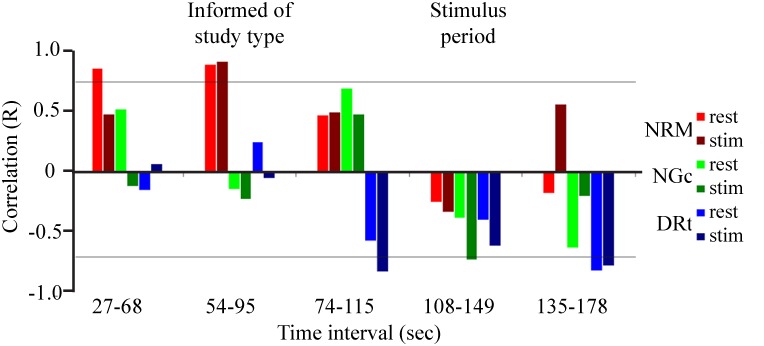
Dynamic variations in the correlation between time-series responses are shown for the regions identified by the SEM analysis as having the strongest relationships (i.e. the best fit). Time intervals spanning 41 seconds were selected throughout the fMRI paradigm, and the correlation was computed to investigate how the relationships evolved. Correlations between the cord DH time series and the NRM (red), NGc (green), and DRt (blue), are shown for the no-stimulation condition (brighter tones), and with a stimulus applied (darker tones). Positive correlations are most notable prior to the stimulation period, and are primarily in the NRM, whereas negative correlations predominate during and after the stimulation period, and are primarily in the DRt. Significant correlations are inferred at |R| ≥ 0.74 (indicated by horizontal lines).

Dynamic correlation analyses (Fig 5) show that the signal variations in the NRM are significantly positively correlated (R ≥ 0.74, p < 0.05) with the signal variations in the cord DH both before and during the periods when participants were informed of the study type. However, the NGc responses are negatively correlated with those in the cord DH during the usual stimulation period, only in runs with stimulation. The DRt time-series responses on the other hand are negatively correlated with those in the cord DH, and only in the periods just before, and after, the usual stimulation period. These correlations reach significance (|R| ≥ 0.74), before the stimulation periods in stimulation runs, and in both study conditions after the usual stimulation period.

## Discussion

The results demonstrate a consistent pattern of BOLD fMRI signal intensity variations across the entire acquisition period, which differs between the no-stimulation condition and the heat stimulation condition, and yet is not a direct response to the stimulation of tissue receptors. Connectivity is demonstrated between seed regions in the C6 segment and a large rostral-caudal extent of the cervical spinal cord, primarily ipsilaterally, in both stimulation and no-stimulation conditions. The BOLD responses in connected voxels have significant variations in the periods when participants were informed of the study type, during the stimulation period, and in the rest period which followed. Similarly, SEM analysis demonstrates relationships between the dorsal horn region at C6, and the NRM, NGc, and DRt in both stimulation and no-stimulation conditions. Dynamic connectivity analyses further demonstrate the relationships between the NRM, NGc and DRt regions with the cord DH, and that the correlations vary with the epoch within the experimental paradigm, in both study conditions. The experimental paradigm was designed to provide long “baseline” conditions for the observation of signal intensity variations and support the objectives of this study. This design is not particularly efficient for detecting BOLD responses to the stimulus because only 30 seconds of each 270 second run (11%) is spent in the stimulation condition [28]. Nonetheless, the results reveal two different components of BOLD signal variations, one which is continuous throughout the time-series acquisition, and the other which is a response to the stimulus.

The spatial patterns of connectivity observed with the seed-to-voxel connectivity analyses are consistent with the resting-state connectivity results shown by Barry et al. [4], in that they show connectivity between dorsal and ventral regions of the spinal cord. Our results are also consistent with features of the rostral-caudal spatial distribution of the resting-state networks detected by Kong et al. [3]. However, there are a number of important differences between our study design, and acquisition techniques, and the methods used by the previous studies. Both of the studies by Barry et al. and Kong et al. employed purely resting-state acquisitions, whereas we intentionally influenced the participants’ expectations and perception related to impending “threat” or “safety”. In addition, their axial slice acquisition methods provided higher temporal resolution and may be more effective at demonstrating some components of the no-stimulation signal that were not captured by our data. Nonetheless, our results have consistent features with those shown in true resting-state studies, and provide evidence of coordinated fluctuations across regions of the spinal cord in the absence of a sensory stimulus. Coordinated responses have also been demonstrated between brainstem and spinal cord regions in response to noxious heat stimuli, mechanical hyperalgesia, and stimulation to induce temporal summation of pain in healthy and fibromyalgia syndrome study groups. SEM analyses have further demonstrated brainstem and spinal cord networks in both healthy participants and people with spinal cord injuries [23, 29–33]. The coordinated responses to noxious stimuli in these studies provide evidence of responses to stimuli as well as descending modulation of the spinal cord. The results from the present study demonstrate connectivity from the C6 region to several adjacent spinal cord segments, spanning more than 24 mm in each direction, within ventral regions, and within dorsal regions. This pattern suggests local communication between these regions, and/or common input from brainstem regions. The connectivity results shown in Fig 2 suggest that this input could be from regions of the rostral or caudal medulla, but the correlation analysis used to infer connectivity does not demonstrate causality or directionality. The correlation analysis method only reveals areas which receive input signaling with temporal components that are in common.

The average time courses across connected voxels (seed-to-voxel connectivity) demonstrate signal intensity variations that are consistent across repeated runs (85 runs in 17 people) and that depend on whether or not the participants were expecting a stimulus, and whether or not a stimulus was applied. The signal intensity variations on the right and left dorsal regions of the spinal cord, shown in Fig 3, demonstrate the responses to participants being informed of the study type, and are quite consistent except on the right side when the stimulus was applied. It is notable that there is no significant response noted on the right side of the spinal cord, when participants were informed that a stimulus would be applied. These observations indicate input signaling to both sides of the spinal cord in response to participants being informed of the study condition, presumably in relation to descending modulation. However, this input is different on the right side of the cord, corresponding to the expected stimulus on the right hand. In addition, BOLD responses corresponding to the timing of the stimulus are detectable on the right side of the spinal cord, only in runs when the stimulus was applied.

The results of SEM analysis to investigate the relationships between signal intensity variations in the spinal cord with variations in the NRM, NGc, and DRt further support these observations. However, while the connectivity analysis reveals regions with similar BOLD time-courses which can be inferred to have similar temporal patterns of input signaling [34], the SEM analysis indicates input signaling and accounts for the fact that regions may receive input from multiple areas. When a stimulus was applied the BOLD signal intensity variations recorded in the cord DH can be explained by a sum of inputs from the periphery (matching the expected BOLD response to the stimulus) as well as from the NRM and DRt. Moreover, the NRM appears to account for signal variations in the cord around the time when the participants were informed of the study type, and the DRt accounts for signal variations just prior to the stimulus and afterward. In the resting state the BOLD signal variations in the cord DH can be explained by a sum of inputs from the NRM, DRt, and the NGc. In this condition the NRM and NGc appear to account for signal variations in the cord DH when the participants were informed of the study type and just prior to the stimulus. The DRt appears to contribute more to the cord DH responses in the later period. This division of the roles of the NRM, NGc, and DRt is supported by the dynamic connectivity analysis which shows a positive correlation between the cord DH and NRM prior to the stimulus, particularly when the participants are informed of the study type. However, the NGc is negatively correlated with the cord DH during the stimulation period. This variation of the effect of the NGc across periods of the paradigm may have resulted in the calculated SEM weighting factor having an overall value that did not reach significance, in runs with stimulation applied. The DRt, on the other hand, is only observed to be negatively correlated with the cord DH, and this occurs in the periods just before the stimulus and later in the paradigm.

The observed relationships between signal intensity variations and the specific period within the stimulation paradigms, and between the responses in the cord DH and the NRM, NGc, and DRt, provide strong evidence that these signal intensity variations are indeed related to neural activity. Moreover, there are significant continuous variations in the spinal cord in relation to the emotional/cognitive state, as well as reactive responses to the stimulus. The RVM (i.e. the NRM and NGc) has been shown in animal studies to provide both descending facilitation and inhibition, and its descending control can vary in relation to effects such as stress, or the region can function as part of a feed-back loop in response to noxious stimulation [11]. As mentioned earlier, the rationale for our study paradigm was based on the fact that the PAG-RVM pathway has been shown to produce analgesia in threatening situations, and the analgesia is inhibited if the environment is perceived to be safe [9, 10]. Our results show how activity in the NRM may have contributed to the activity observed in the spinal cord DH when the participants were informed of the study type (no-stimulation or stimulation), and in the interval prior to the application of the stimulus, whereas the NGc appeared to contribute more to the cord input signaling during stimulation. The relationships between the cord DH activity and the NRM and NGc activity prior to the stimulus are consistent with the expected role of the RVM in providing modulation of spinal cord responses in response to noxious stimulation, and continuous modulation in relation to the emotional/cognitive state such as with the expectation of threat or safety. The DRt has been shown in animal studies to provide input to the spinal cord to facilitate pain responses with both tonic signaling and as part of a positive feed-back loop [35]. Negative correlations between DRt and cord DH BOLD signal variations, and the negative SEM weighting factors for the DRt, suggest that the cord DH received less input when the DRt received more input. This may reflect that the DRt received inhibitory input to diminish its pro-nociceptive action when a stimulus was anticipated, while the stimulus was applied, and again afterward when no stimulus was expected. This is consistent with the DRt providing both continuous (tonic) and reactive contributions to descending modulation of spinal cord nociceptive responses, as expected from animal studies.

Our results demonstrate that systematic BOLD variations occur in the spinal cord and brainstem, in the absence of a sensory stimulus, and in the periods before and after a noxious stimulus. The stimulus involved both noxious heat and touch sensations on the right hand, and we expect that the observed responses depend strongly on the prior training the participants received so that they were very familiar with the sensations produced by the stimulus, and the timing of when it was applied during each fMRI acquisition. This approach has enabled us to observe BOLD signal intensity variations that are related to the emotional/cognitive state, are continuous, and likely reflect descending regulation of spinal cord activity. Our results also provide evidence that the continuous and reactive responses in the spinal cord are caused, at least in part, by input signaling from the NRM, NGc, and DRt. Several brain fMRI studies have demonstrated responses related to anticipation of thermal pain on the hand, in the anterior cingulate cortex (ACC), anterior insular cortex (AI), and regions of the prefrontal cortex [36–39]. In animal models the ACC has been shown to influence descending pain modulation by contributing to pain facilitation, or to analgesic effects, depending on the study conditions [40–42]. One fMRI study also demonstrated responses related to anticipation of thermal pain in the periaqueductal gray (PAG) region, and proposed that this is likely related to descending modulation [43]. However, it is a key point of the current study that we have also observed brainstem and spinal cord responses when no painful stimulus was expected. We suspect that the continuous modulation of spinal cord neurons not only gives rise to the observed variations in the absence of a stimulus but may also play a role in emotional/cognitive modulation of pain, such as variations in pain sensitivity with attention and mood, and phenomena such as placebo, nocebo, anticipation of pain, and pain catastrophizing [36, 43–46]. However, further study is needed to investigate these questions.

### Limitations

This study contributes to our understanding of BOLD signal fluctuations in the spinal cord in the absence of a sensory/pain stimulus, and in the periods before and after a stimulus is applied. However, the “no-stimulation” runs in the present study involved instructing the participant that a stimulus would not be applied, one minute after the start of the run, and the responses observed are expected to depend on the participants being very familiar with the study paradigm and the painful stimulus. The results therefore do not represent a true “resting-state” and they do not necessarily demonstrate the source of BOLD signal fluctuations which have been reported in the resting-state [2–4], The results show only that emotional/cognitive factors could contribute to resting-state signal variation in the spinal cord. A limitation of our fMRI study design is the lack of an innocuous sensation control condition, which may have enabled us to extend our findings and confirm whether the observed responses are related to pain specifically. This limitation was imposed by our need to maintain the fMRI sessions at a reasonable duration to avoid participant fatigue and discomfort.

Physiological noise and motion effects present challenges for fMRI studies of the brainstem and spinal cord, and these effects may have interfered with detection of BOLD responses [12, 47, 48]. However, the fMRI paradigm used in the present study was designed to exploit the fact that physiological noise and motion are incoherent across repeated fMRI runs, and analysis methods have been developed to reduce these effects in the data from each run. The time-series responses shown are the average of 85 runs in 17 participants and demonstrate predominantly the coherent BOLD responses across repeated runs. Connectivity and SEM analyses similarly show the consistent effects across repeated runs, but nonetheless the sensitivity may still have been reduced by the effects of movement and physiological noise.
